# Development and validation of cultural competence assessment tool for healthcare professionals, India

**DOI:** 10.3389/fpubh.2022.919386

**Published:** 2022-08-23

**Authors:** Parvathy Balachandran, Vineetha Karuveettil, Chandrashekar Janakiram

**Affiliations:** Department of Public Health Dentistry, Amrita School of Dentistry, Amrita Vishwa Vidyapeetham, Kochi, India

**Keywords:** cultural competency, cultural diversity, India, health personnel, healthcare system, patient care

## Abstract

Culture influences an individual's perception of “health” and “sickness”. Therefore, cultural competence assessment of healthcare professionals is very important. Existing assessment scales have limited application in India due to the nation's rich cultural diversity and heterogeneous healthcare streams. This study was undertaken to develop and validate a cultural competence assessment tool for healthcare professionals in India. A cross-sectional study using convenience sampling was conducted following all standard steps among 290 healthcare professionals in India. Item reduction was followed by estimation of validity and reliability. Responses were recorded on a five-point Likert scale, ranging from strongly disagree to strongly agree. The resultant tool, named Cultural Competence Assessment Tool–India (CCT-I) showed an acceptable internal consistency (Cronbach's alpha =0.734). Inter-rater agreement was 81.43%. Face, content, and construct validity were demonstrated. There was no statistically significant difference in cultural competence between the healthcare streams based on years of clinical experience. There was statistically significant difference between streams of healthcare (*p*-value =0.009) and also between dentistry and Ayurveda groups (*p*-value = 0.003). This comprehensive tool can be used as the first step toward designing cultural competence training of healthcare manpower and the establishment of culturally sensitive healthcare organizations.

## Introduction

Health equity is the cornerstone of community-focused health interventions and aims to improve the wellbeing of each individual in the community. However, achieving health equity is a challenging process as it comprises complex interactions among healthcare demand, supply, accessibility, and utilization, which, in turn, depend on the social determinants of health. These are conditions in places where people live, learn, work, and play that affect a wide range of health and quality-of-life risks and outcomes (1) like social norms, education, job, income, and gender roles, which widen the health disparities in the community.

Among the social determinants of health, culture is the most important yet often neglected factor in healthcare. Culture refers to “integrated patterns of human behavior that include language, thoughts, communications, actions, customs, beliefs, values, and institutions of racial, ethnic, religious, or social groups” (2). It is a fundamental characteristic of a community that has a deep-rooted impact on the life of an individual, his/her belief systems, and attitude, including one's perception of “health” and “sickness”. Culture determines the presentation and interpretation of disease symptoms, health-seeking behavior, and treatment outcomes. Therefore, a healthcare system that understands and accepts the existence of different cultural groups among healthcare seekers is a huge step toward a successful health service delivery system (3). It also highlights the importance of cultural competence development of healthcare manpower.

Competence implies “having the capacity to function effectively” (4). Cultural competence is “a set of congruent behaviors, attitudes, and policies that come together in a system, agency, or among professionals and enable that system, agency, or those professionals to work effectively in cross-cultural situations” (4). This description of cultural competence gives insights into a culturally competent healthcare system, that is, “one that acknowledges and incorporates—at all levels—the importance of culture, assessment of cross-cultural relations, vigilance toward the dynamics that result from cultural differences, expansion of cultural knowledge, and adaptation of services to meet culturally unique needs” (5).

This dimension of healthcare is highly relevant in the current COVID-19 pandemic, where the literature shows that cultural diversity is an important challenge to equitable distribution of healthcare services and accessibility (6). Efforts to enhance cultural competence in the healthcare system have made a significant impact in the United States, like the Initiative to Eliminate Racial and Ethnic Disparities in Health, which implemented the National Culturally and Linguistically Appropriate Services Standards and delivered culturally appropriate influenza immunization in addition to establishing grants and community networks program centers to reduce cultural disparities in healthcare (7).

Cultural competence enables a healthcare provider to go beyond the pathophysiological knowledge of disease (8). A culturally competent healthcare worker understands patient's perspective of health and illness, has improved healthcare provider–seeker interaction, overcomes language barriers, and increases the quality of care, thereby resulting in a positive treatment outcome (9). Studies have shown that culturally relevant interventions improved health outcomes related to sexually transmitted diseases, type II diabetes (10, 11), and drug addiction (12). Similarly, culturally sensitive healthcare services like use of bilingual community health workers have improved culturally diverse patients” acceptance of cancer screening and health monitoring (13).

“Culture” being a subjective concept is often used synonymously with socioeconomic status, leading to underestimation of the role of culture in a person's life (5). Another challenge, particularly in a vast and diverse nation like India, is the existence of many cultures along with their equally numerous subcultures. Each culture and subculture is unique, and a broad stereotyping of patients by preconceived notions can result in unintentional harms. Scarcity of time and resources, reluctance, or failed efforts in recognizing the cultural impacts on health, and incompetent leadership to highlight the importance of culture are other limitations of cultural competence in healthcare.

Cultural competence development of healthcare professionals is an issue of prime importance. It has gained priority in Western countries; however, it is still an unexplored area in the Indian setting. The first step toward the development of this skill is its assessment. Although there are many cultural competence assessment tools available globally, applicability of such tools varies widely based on the cultural environment of each country. In addition, India has multiple healthcare streams, like allopathy, Ayurveda, homeopathy, Siddha, Unani, and naturopathy, where the perspective of disease, its causes, and treatment approaches are highly heterogeneous. Therefore, to assess the cultural competence of healthcare professionals in India, there is a necessity for an assessment tool that applies to the healthcare system, which is influenced by a multitude of cultures, belief systems, healthcare streams, and social norms. Currently, there is no available assessment tool that is specifically adapted to the Indian setting. The present study was therefore designed to address this significant gap in the literature by developing a cultural competence assessment scale customized for Indian healthcare professionals.

## Methods

This cross-sectional study using convenience sampling for selection of participants was undertaken from March 2021 to October 2021. Prior to the conduct of the study, ethical approval was obtained from the Institutional Ethics Committee of Amrita Institute of Medical Sciences, India (ECASM-AIMS-2021-171, date: 23-02-2021). Due to the COVID-19 pandemic, communications related to the study were undertaken through email and online platforms using Google Forms. Description of the study was provided to all stakeholders through email and Google Forms, and informed consent was obtained from them in the same manner.

The proposed cultural competence assessment scale for Indian healthcare professionals was to be developed in the form of a questionnaire with the following features:

- It has the ability to measure cultural competence through participants” responses to the questions.
- Being a novel instrument, it can assess the cultural competence level of healthcare professionals from different healthcare streams in India, a unique feature of the proposed scale as the various healthcare streams often have conflicting theories and approaches. We focused on the patient–caregiver interaction, which is the most important aspect of any treatment, irrespective of the healthcare stream.
- It includes nursing professionals of different healthcare streams as they play a profound role.
- It comprises questions that cover the entire spectrum of cultural competence.
- It is amenable to statistical analysis.
- It has good psychometric properties with satisfactory reliability and validity.

Only healthcare professionals belonging to medical, dental, Ayurveda, nursing, and homeopathy streams, with at least 3 years of clinical experience after their graduation, were included in the study. The nursing group also included dental assistants, Ayurveda and homeopathic nurses, and those with auxiliary nurse midwife (ANM) and general nursing and midwifery (GNM) qualifications. Eligible healthcare professionals who were unwilling to participate were excluded from the study. Healthcare graduates who had changed their careers to other streams, like bioinformatics and insurance sectors, were also excluded from the study.

Questionnaire development was performed in three phases:

- Phase of item development involving the identification of domains, item generation, and content validation;
- Phase of scale development involving pretesting the developed questionnaire using cognitive interviews and exploratory factor analysis;
- Phase of scale evaluation consisting of tests of reliability and validity.

Domains were identified and developed by literature review. The distinction between the domains was ensured by expert validation. A pilot version of the questionnaire with 159 items was developed using a deductive approach through extensive literature review. After the removal of overlapping and redundant items, it had seven domains and 43 items in total. For validation of domains and items, two Google Forms were designed: one for domains and the other for items.

The degree of relevance of each domain and item to measure the cultural competence of healthcare professionals was recorded by a three-point Likert scale and four-point Likert scale, respectively. Google Forms also comprised the participant information sheet and certificate of consent. These Google Forms were administered to an expert committee of five members. Based on their responses, kappa scores of agreements were calculated. The resultant questionnaire comprised six domains and 35 items.

**Figure 1:**
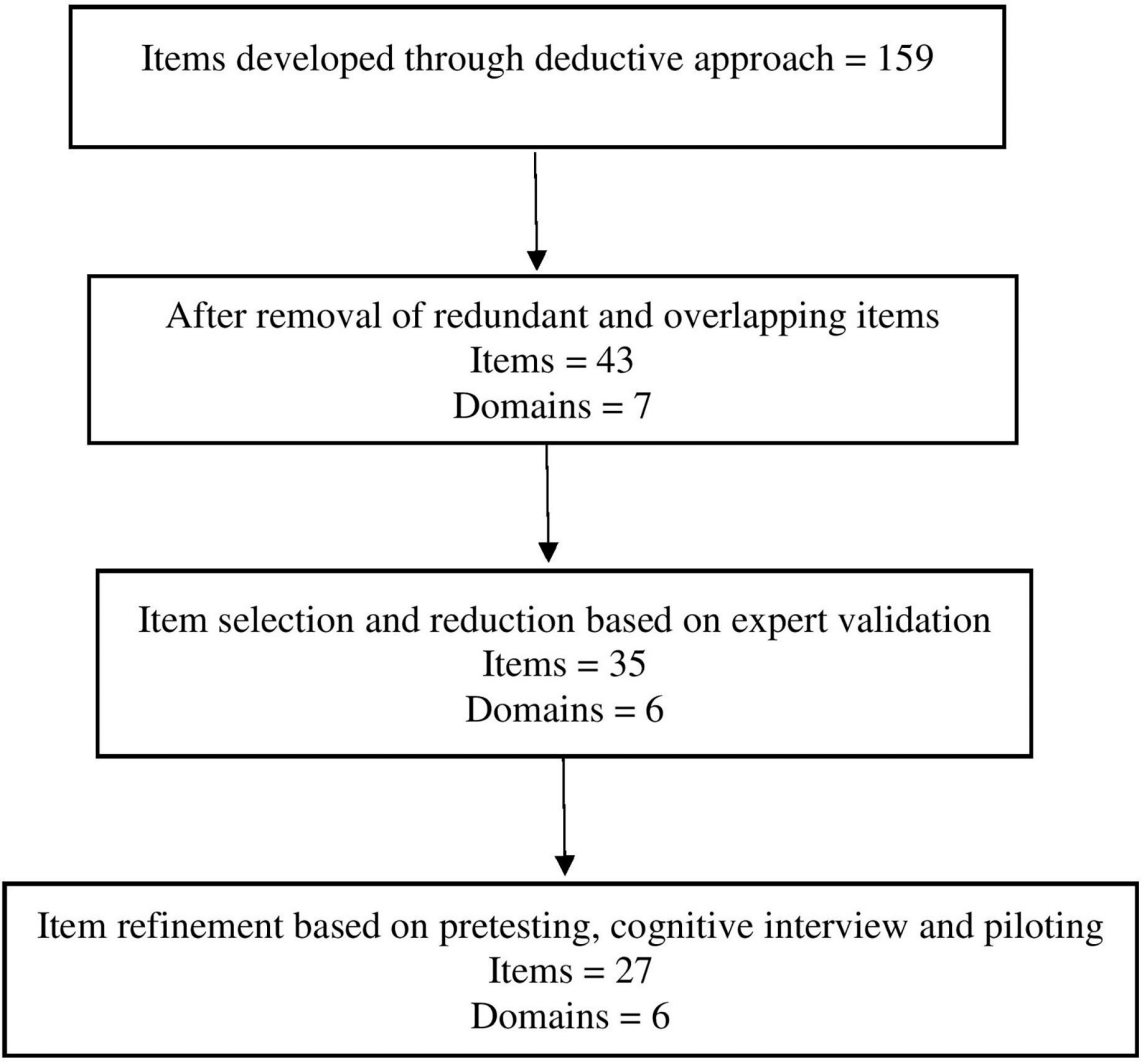
Item generation and item reduction.

Cognitive interviews were carried out among 10 participants to ensure the quality and accuracy of the questionnaire and to identify sources of response errors before administering it to final users. We used the “think aloud” approach for five participants and the “verbal probing approach” for five participants. The tool was then piloted on a sample of 30 participants. Internal consistency of the cultural competence questionnaire was determined using Cronbach's alpha. Test–retest reliability was assessed among the 30 participants after 2 weeks by using the intraclass correlation coefficient. Based on the test–retest reliability score, eight items were eliminated. The resultant tool had six domains and 27 items (Figure 1).

The sample size for scale development is based on the rule of thumb of at least 10 participants for each scale item, with a 10:1 respondent-to-item ratio (14). Therefore, the minimum sample size of the pilot study was estimated to be 270, with at least 54 participants from each healthcare stream to ensure uniform participation. The content validity index (CVI) is the most commonly used content validity approach. In estimating the scale-level-CVI, two methods were employed: the average-CVI (S-CVI/Ave) and universal agreement (UA) among experts (S-CVI/UA).

The reliability of inter-rater agreement was estimated by using Fleiss kappa. Known group validity was assessed by comparing scores among different healthcare streams. Based on the responses, construct validity was assessed using exploratory factor analysis (EFA). This step was carried out to reduce the number of items and to ensure that the developed tool successfully measured the cultural competence of healthcare professionals. Based on the EFA, one item was removed. Following this, the floor and ceiling effects were determined.

**Figure 2:**
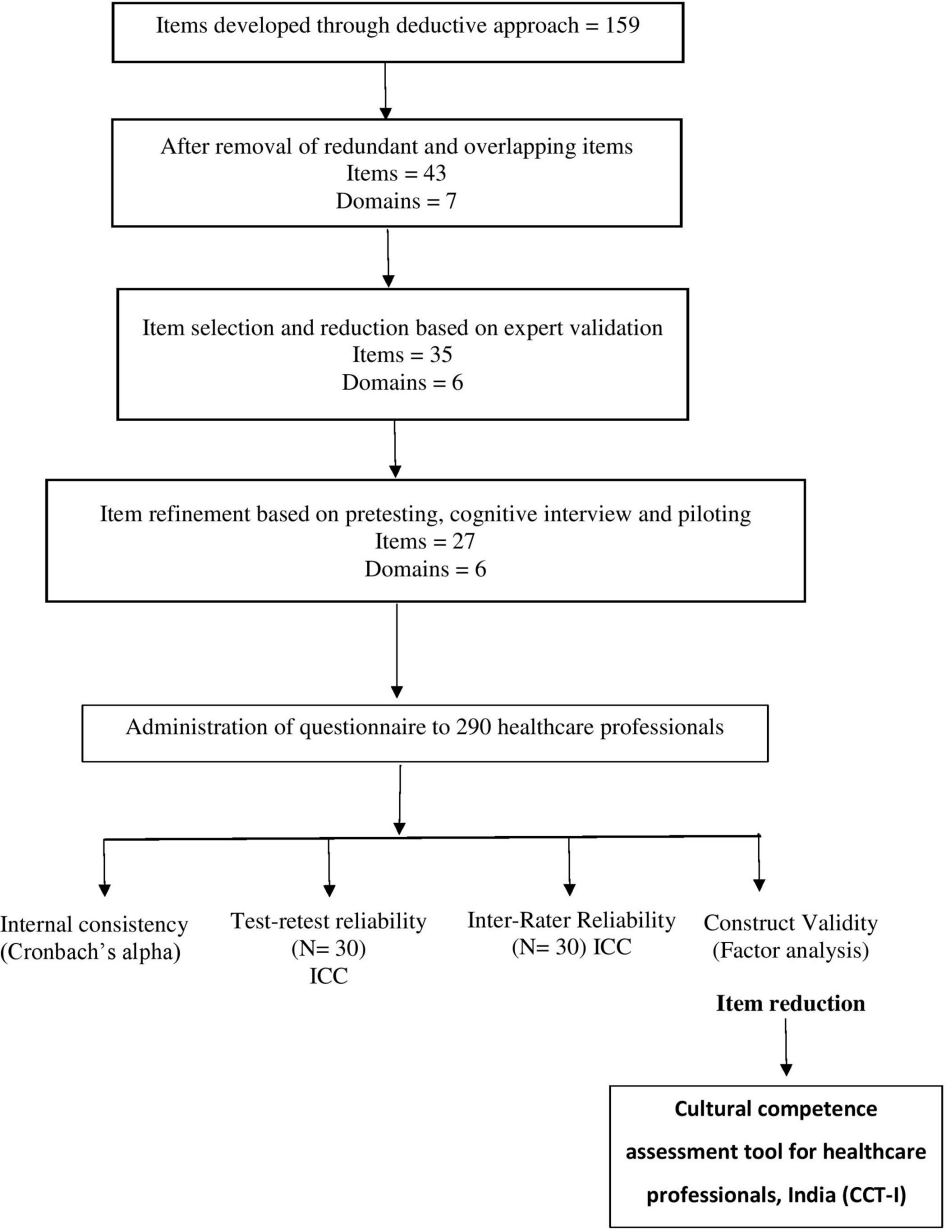
Flow chart showing the steps in the development of the new tool.

The final tool to assess the cultural competence of healthcare professionals in India was named Cultural Competence assessment Tool–India (CCT-I). It had six domains and 26 items (Figure 2). Participants' responses were recorded on a five-point Likert scale with the following scoring criteria: 1= strongly disagree, 2= disagree, 3= neutral, 4= agree, and 5=strongly disagree. To minimize social desirability bias, six items were negative worded, and hence, their scores were inversed during analysis. Standardization of scores was based on percentile rank of scores. Statistical analysis was performed using IBM SPSS Statistics for Windows, version 23 (IBM Corp., Armonk, N.Y., USA).

## Results

### Sample description

A total of 290 participants completed the survey, with a response rate of 86.05%. The mean age of the participants was 35.09 ± 9.85 years. A maximum number of the participants were from nursing (*n* = 67), followed by dentistry (*n* = 58), homeopathy (*n* = 56), Ayurveda (*n* = 55), and medicine (*n* = 54). It was found that the majority of participants had <5 years of clinical experience (35.9%) (Table 1). The Shapiro–Wilk test was used to test normality as the sample size was more than 50. Since the data were skewed, median and interquartile range were estimated.

**Table 1:** Characteristics of the study population.

**Parameters**	**Result**
Age in years	35.09 ± 9.85 years
**Healthcare system**	
Medicine	54 (18.6%)
Dentistry	58 (20%)
Ayurveda	55 (19%)
Homeopathy	56 (19.3%)
Nursing	67 (23.1%)
**Years of clinical experience**	
3– 5 years	104 (35.9%)
5– 10 years	81 (27.9%)
10–15 years	48 (16.6%)
15–20 years	12 (4.1%)
20–25 years	16 (5.5%)
More than 25 years	29 (10.0%)

For the domain of cultural awareness, the median and interquartile range is 25 (22–27). The median and interquartile range of the cultural sensitivity domain is reported to be 12 (11–14), 14 (13–16) for the cultural knowledge domain, 10 (9–11) for the cultural skills domain, 14 (13–15) for the cultural behavior domain, and 16 (14–16) for cultural desire domain. The overall median and interquartile range of the cultural competence scale is 91 (84–96) (Supplementary Table 1).

### Floor and ceiling effect calculation

The floor and ceiling effects for the cultural competence scale were satisfactory. The overall floor effect was 11.38%, and the ceiling effect was 12.41%. The floor effect for each domain ranged from 7.58 to 14.14%, and the ceiling effect ranged from 5.52 to 24.48%. When considering individual domains, except for cultural knowledge, which had a ceiling effect of 24.8%, other domains maintained the required scores (Supplementary Table 2).

### Reliability estimates

Reliability analyses were performed on the 35-item questionnaire in phase I (Supplementary Table 3) and the 27-item questionnaire in phase II. Test–retest reliability after item reduction was measured for 27 items. The overall intraclass correlation coefficient was estimated as 0.767, indicating satisfactory stability (Supplementary Table 4). Item-wise test–retest reliability was also performed, and the intraclass correlation coefficient ranged between 0.566 and 0.822. Internal consistency of the 35-item questionnaire was estimated using Cronbach's alpha (Supplementary Table 5), and eight items were removed. Overall internal consistency using Cronbach's alpha for the 27-item questionnaire was estimated to be 0.700, which was acceptable (Supplementary Table 6). After removal of one item with a lower eigenvalue, overall internal consistency improved to 0.734, which was acceptable (Supplementary Table 7).

### Validity estimates

The face validity and content validity of the proposed tool were determined by assessing its content during the expert reviews (Supplementary Tables 8, 9) and peer reviews in the phase of item development. After the removal of one domain (domain 7) and two items, for domains, S-CVI/Ave was 0.92 and S-CVI/UA was 0.5. Fleiss kappa for domains yielded a fair score of 0.67, and the overall agreement was 83.30% (Supplementary Table 10). For items, the average-CVI (S-CVI/Ave) was estimated to be 0.87 and S-CVI/UA was 0.58. Fleiss kappa was found to be 0.25, and the score-combined kappa was 0.72. Agreement among the raters was 81.43% (Supplementary Table 11).

Construct validity was established by using exploratory factor analysis (EFA) (Supplementary Table 12) with principal component analysis (PCA) and varimax rotation in the phase of scale evaluation. Initial factor extraction with PCA yielded 49.35% as cumulative percentage variance explained by the tool (Supplementary Table 13). Minimum loading for an item with a factor is expected to be 0.35 (15). Then, one item was found to have a factor loading <0.35 and was removed from the tool; six items showed a cross-loading effect. However, these items were relevant to the tool in the assessment and were therefore retained. Factor analysis was performed for the remaining 26 items. The cumulative percentage variance explained by the tool improved to 50.36% (Supplementary Table 14).

The final factor analysis resulted in six domains, with a total of 26 items being established. The absolute loading value gives the relationship between the item and the concept of cultural competence. Only one item showed a low loading value, and six items showed a cross-loading effect. Since the items were relevant for the assessment of cultural competence, these items were retained in the tool.

Known group validity was estimated using the Kruskal–Wallis test for years of experience and stream of healthcare. There was no statistically significant difference between the groups for years of experience (Table 2A). However, there was statistically significant difference between the groups in relation to the stream of healthcare (*p*-value = 0.009). Mean and standard deviation was highest for the Ayurveda group (93.38 ± 8.39), followed by homeopathy, medicine, nursing, and dentistry, respectively (Table 2B). The difference in cultural competence between dentistry and Ayurveda groups was found to be statistically significant (*p*-value = 0.003) (Table 3).

**Table 2A:** Known group validity. Known group validity based on years of experience: Kruskal–Wallis test.

**Years of experience**	**Mean ±SD**	**Median**	**IQR**			**Chi-Square**	**df**	**Sig**.
			**Q1**	**Q2**	**Q3**			
<5 years	88.63 ± 8.74	88	82	88	95	6.96	5	0.223
5–10 years	90.96 ± 7.75	92	84.5	92	96			
10–15 years	92.17 ± 7.13	92.5	88.25	92.5	97.75			
15–20 years	92.00 ± 7.75	90	86	90	99			
20–25 years	91.88 ± 12.76	92	85	92	96.75			
More than 25 years	89.97 ± 7.78	91	82.5	91	95.50			

p-value ≤0.05 is considered statistically significant.

IQR, interquartile range.

**Table 2B:** Known group validity based on healthcare streams.

**Stream**	**Mean ±SD**	**Median**	**IQR**			**Chi-Square**	**df**	**Sig**.
			**Q1**	**Q2**	**Q3**			
Medicine	90.15 ± 7.53	91.5	85	91.5	95	13.61	4	0.009^*^
Dentistry	87.28 ± 7.70	86.5	81	86.5	94			
Ayurveda	93.38 ± 8.39	92	88	92	99			
Homeopathy	91.23 ± 8.19	91	84.25	91	96.75			
Nursing	89.82 ± 9.07	90	83	90	96			

* p-value ≤ 0.05.

**Table 3:** Known group validity based on healthcare streams.

**Stream**	**Median 1**	**Median 2**	**Sig**.
Dentistry-medicine	86.5	91.5	0.632
Dentistry-nursing	86.5	90	0.939
Dentistry-homeopathy	86.5	91	0.158
Dentistry-ayurveda	86.5	92	0.003^*^
Medicine-nursing	91.5	90	1.000
Medicine-homeopathy	91.5	91	1.000
Medicine-ayurveda	91.5	92	0.926
Nursing-homeopathy	90	91	1.000
Nursing-ayurveda	90	92	0.403
Homeopathy-ayurveda	91	92	1.000

* p-value ≤ 0.05.

### Standardization of scores

Cultural competence scores of the range 26 to 84 were considered as low (0 to 24th percentile). Scores from 85 to 96 were considered as average cultural competence (25th to 75th percentile), and scores from 97 to 130 were considered as high cultural competence (76th to 100th percentile).

## Discussion

This article reports on the field test of the CCT-I as part of the instrument development process. Existing cultural competence assessment scales had limited applications in the Indian setting due to the country's diversity of cultures and healing systems. Moreover, existing scales varied widely in their interpretation of “culture,” “cultural competence,” and therefore the assessments (15). Most of these tools were group-specific, focusing only on nurses, pharmacists, and dentists, thereby curtailing their application in the comprehensive assessment of cultural competence at the organization or national level (16). On this account, there was a compelling need to develop and validate a novel assessment tool that is focused on assessing the wide spectrum of cross-cultural competence, irrespective of the healthcare stream.

The present study was performed in accordance with the standard steps of scale development and validation (14) and the COnsensus-based Standards for the selection of health status Measurement INstruments (COSMIN) (17). The proposed scale was developed as a self-reported assessment tool since it is more appropriate for expressing one's attitude, beliefs, and behaviors. Although there are drawbacks like social desirability bias, response bias, and lack of opportunity to clarify the respondent's doubts, the current tool was designed as a self-reporting questionnaire because respondents were accustomed to the issues in question and the information they give in self-report questionnaires tends to be more accurate (18).

Domains and items constituting the CCT-I scale were derived by literature review and consensus. Domains were validated by a committee of six experts and items by a committee of five experts. For a scale to be considered as having excellent content validity, all its items should have an I-CVI score of 1 (19). As reported, the I-CVI was acceptable after elimination of two items; four items which showed low I-CVI scores of 0.6 were revised. The remaining items showed scores ranging from 0.80 to 1. Such modifications were made in similar studies like the development of an instrument to measure patient-centered communication (20). In this study, items that scored below 0.7 were eliminated and that showed scores between 0.7 and 0.79 were revised. This shows that all items in the tool were conceptually relevant and appropriate to assess the cultural competence of healthcare professionals in India.

In this study, S-CVI/UA was 0.43 for domains and 0.47 for items. S-CVI/Ave for domains was 0.86 for domains and 0.67 for items. After the removal of one domain (domain 7) and two items (items 10 and 37), S-CVI/UA for domains was found to be 0.5 for domains and 0.58 for items. S-CVI/Ave improved to 0.92 for domains and 0.87 for items. S-CVI/Ave >0.90 denotes excellent score for scale-level content validity (19). According to Lynn's criteria for item acceptability, excellent content validity is characterized by I-CVIs of 0.78 or higher (21). Similarly, the S-CVI/UA value of 0.8, as per the conservative requirement of 100% agreement at the item level for at least 80% of items (22–24), and S-CVI/Ave value of 0.9 or higher (25), denote excellent content validity. These benchmarks show that the CCT-I scale has satisfactory content validity.

Chance agreement is an issue of concern in validation by assessors (26); hence, kappa statistics was also computed. The combined kappa value for the developed tool was 0.72, thereby indicating a good score (20). Overall percentage agreement among the expert judges improved to 83.30% for domains and 81.43% for items. This is in accordance with recommendations that an agreement of 80% or higher is considered ideal for tool development (20).

The next step in tool development was pretesting the tool *via* cognitive interviews to ensure that the target population clearly understands the domains and items (14). Think aloud is a method of cognitive interview in which the participants are given the opportunity to verbalize their thought process as they answer the items. Verbal probing is the alternative procedure of cognitive interview, where the interviewer probes the interviewee with additional questions to elicit further information on the items of the tool (27). Since both are unique in their approach and technique, we conducted cognitive interviews for five participants using the think aloud process and another five participants using the verbal probing method.

Typographical and grammatical errors were identified through cognitive interviews. It was recommended to revise certain items to make the assessment tool suited for the Indian scenario. A change in the rating of the Likert scale was suggested by three interviewees due to the difficulty in differentiating between strongly agree and agree and likewise between strongly disagree and disagree. However, we did not change the five-point Likert scale as it was befitting our questionnaire. Another suggestion was to rephrase some of the items to reduce possible social desirability bias. To resolve this issue, some of the items were negative worded, and hence, their scores were inversed during analysis. There was an overall consensus on the length of the questionnaire and time taken to answer.

Another component of content validation is the identification of floor and ceiling (F/C) effects. The floor effect implies that the items are hard to understand, while the ceiling effect means that items are easy to understand. In some studies (28, 29), 5 or 10% is considered the benchmark for the F/C effect. However, in the majority of studies, a score of ≥15% is considered to have a significant F/C effect. We also followed the 15% criterion in this study. The domain corresponding to cultural knowledge showed a ceiling effect of 24.48%. However, the overall F/C effect score of the developed tool was 11.38 and 12.41%, respectively, which was acceptable.

We used test–retest reliability and Cronbach's alpha to determine the reliability of the tool. For the test–retest reliability (coefficient of stability) approach, the assessment tool was administered to 30 participants as Google Forms contained 35 items. The suggested gap between the test and retest is 2 weeks (30), which was followed in this study. The intraclass correlation coefficient was used to determine the reliability of the scale. Items that showed values closer to 0 indicated low reliability (14). Internal consistency of the developed tool was assessed by Cronbach's alpha. An alpha coefficient of 0.70 is an acceptable threshold for reliability (14). A benchmark of 0.70 for Cronbach's alpha was used in studies associated with the development of similar scales like the cultural capacity scale and validation of its Arabic version (31). Based on the intraclass correlation coefficient and Cronbach's alpha, eight items were removed. The resultant questionnaire had 27 items.

Factor analysis required a sample size of at least 10 participants for each scale item (14), with a 10:1 respondent-to-item ratio, resulting in 270 samples. To achieve equal response rate from the five streams of healthcare, we obtained a minimum of 54 samples from each group through convenience sampling. This ensured the applicability of the tool to the various healthcare streams in India. An assessment tool should be a parsimonious representation of the entire spectrum of the concept of interest. Our efforts were to develop a cultural competence assessment questionnaire for healthcare professionals in India with items that were unique to the domain represented, thereby minimizing overlaps. This constitutes the property of construct validity, which was carried out by using exploratory factor analysis (EFA) with principal component analysis (PCA) and varimax rotation.

EFA involves deciding on a factoring method, choosing a rotation procedure, and interpreting the results. The number of factors that are retained during the process of EFA is decided by eigenvalues of each factor (32). According to the Kaiser–Guttman rule, all factors for which the eigenvalue is >1.0 should be retained (33). In the developed tool, the eigenvalue was >1. This is in congruent with previous studies involving EFA (34, 35).

The number of items that distinctly measured a particular domain was estimated through factor loading using PCA. Initial factor extraction with PCA yielded 49.35% as cumulative percentage variance explained by the tool. PCA was followed by varimax rotation, which is the most common orthogonal rotation method (36). Factor loading was used on 27 items. The factor loading matrix that showed a higher value implied a strong relation between the factor and the item (37), and a value of 0.35 is assumed to be the minimum loading value (38). After removal of the item with a factor loading value <0.35, factor analysis yielded a cumulative variance percentage of 50.36%.

Cultural competence of healthcare professionals implies their ability to successfully interact with and treat patients from diverse cultural backgrounds. Our validity of the hypothesis was that the Indian healthcare environment was unique due to multiple healthcare streams with often conflicting principles and practices. Thus, the difference of cultural competence with respect to healthcare streams and years of clinical experience was tested for their significance as a further step to ensure known group validity. The difference in cultural competence based on years of clinical experience, as estimated by the Kruskal–Wallis test, was not statistically significant. This finding is similar to the result of a study among nurses in Bangkok, where it was found that nursing experience did not have a significant correlation with cultural competence (39). Another study conducted among registered nurses and psychiatric unit healthcare workers also showed that experience alone does not have a significant effect on the cultural competence level (40).

There was statistically significant difference in cultural competence based on the healthcare stream. Currently, there are no available studies comparing the cultural competence of healthcare workers from different healthcare streams. Multiple assessment tools, priority of transcultural nursing in curriculum, and various cultural models in nursing prove that cultural competence is given an important role in nursing than in other healthcare professions (40–49). Analysis of the various healthcare streams yielded a statistically significant difference in cultural competence between dentistry and Ayurveda.

## Strengths and limitations

The Cultural Competence Assessment Tool–India (CCT-I) is a novel attempt specifically focused on the Indian healthcare environment, where culture plays a deep-rooted effect on health. Since cultural competence development is a dynamic process, we have covered its various aspects, making this a comprehensive assessment tool that is applicable to different healthcare systems being practiced in India. Currently, India is witnessing a paradigm shift to patient-centered healthcare, which is a harbinger for the establishment of a culturally sensitive healthcare system and culturally sensitive health workers. The first step toward this is the assessment of the existing cultural competence level of organizations and its manpower for which the developed tool is appropriate. A major strength of this tool is the broad coverage of the concept of “cultural competence”. Existing assessment tools confine mostly to two or three domains, while our tool covers six domains, thereby helping in a comprehensive assessment.

However, our study has a few limitations. The main limitation of the developed questionnaire is the subjective nature of the concept of “culture.” Consequently, we did not undertake focus group discussion for domain and item preparation as it will be impractical to achieve a saturation of viewpoints on this vast topic. Moreover, the COVID-19 pandemic was a barrier to our communications throughout this study. Another drawback was the delicate distinction between the various aspects of the spectrum of cultural competence as depicted by the domains of the tool. In addition, the response rate of validators and participants for cognitive interview was low. Although precautions were taken to overcome social desirability bias, the sensitive nature of the topic may limit its elimination.

## Policy implication

The past decade has witnessed tremendous improvement in the healthcare system owing to advancements in technology and research. However, the disease burden in the country remains unchanged, particularly among the underprivileged and underrepresented communities. This dilemma in the Indian health system highlights the urgency to identify and resolve barriers to the “health for All” concept. Multiple factors like poverty, ignorance, healthcare accessibility, social norms, and gender roles challenge our health system. An insight into these multifarious barriers shows that the majority of these factors are based on the cultural beliefs of the people. Culture plays a crucial role in the lifestyle and practices of an individual in India. However, this vital determinant is overlooked at the organizational and policymaking levels. This neglect eventually cripples the system because the benefits of medical advancements will be channeled solely to the “elite” group.

The alarming contribution of India to the global burden of disease emphasizes the critical need of integrating cultural competence training into the healthcare curriculum. Cultural competence orientation of healthcare trainees from the time they start their clinical postings is an effective strategy in enhancing healthcare accessibility and utilization, thereby downsizing the “cultural gap” existing in the Indian healthcare setting. The Cultural Competence Assessment Tool–India (CCT-I) scale that is developed through this study is an important landmark in such a scenario. This is because the assessment of healthcare workforce, irrespective of their healthcare streams and trainings, will be a harbinger of reforms in the healthcare setting like development and implementation of cultural competence enhancement programs, incorporation of such training programs in the healthcare undergraduate courses, and establishing patient-centered, culturally competent healthcare facilities. This orientation of healthcare is being witnessed in Western countries where accrediting boards and the higher education system have started making cultural competence training a mandatory exercise (45, 50–52).

Studies have shown that cultural competence of healthcare personnel bears positive outcomes in treatment and communications (41, 50, 51, 53–55). It helps mitigate the longstanding mistrust of communities in treatments and overcome the social ostracism commonly seen in diseases like leprosy, skin diseases, depression, and epilepsy. Strengthening cultural competence of healthcare professionals helps patients communicate better regarding their concerns, expectations, and fears, thereby enabling the care providers to incorporate their decisions in treatment. To achieve this patient-centered approach, it is essential for the policymakers to understand the importance of cultural competence in healthcare. Quantifying it based on a tool like CCT-I, which focuses on the Indian context, is an ideal step to capture the attention of policymaking circles for this purpose.

## Research implication

The concept of cultural competence is a less ventured domain in India. Although there are multiple studies on culture and its implications on a person's life, there is scarcity of the literature in the context of healthcare. India is a land of many cultures and subcultures, and each of these has manifold beliefs and practices related to health and healing. Moreover, some indigenous healing systems in India are firmly based on cultural beliefs. Often, these multiple healing systems and patient beliefs are conflicting, thereby delaying treatments resulting in morbidity and mortality.

Therefore, the scope of research on the various culturally rooted health practices is vast. Moreover, studies on the prevalence of culture-bound syndromes and cultural practices that influence health are inadequate in India. This study also warrants future cultural competence assessment studies in the Indian setting using the CCT-I scale along with qualitative approaches like patient simulation for improved knowledge in this domain. The developed CCT-I is a steppingstone to the identification of the cultural impact in healthcare, which subsequently leads to widening the research prospects in this field.

## Conclusion

This study resulted in the development of a novel cultural competence assessment tool specifically designed for Indian healthcare professionals. The tool, named Cultural Competence assessment Tool–India (CCT-I), consists of six domains and 26 items. This comprehensive tool can be used to assess the cultural competence level of healthcare professionals as the first step toward designing cultural competence training for healthcare manpower and the establishment of culturally sensitive healthcare organizations in India.

## Data availability statement

The datasets presented in this study can be found in online repositories. The names of the repository/repositories and accession number(s) can be found in the article/Supplementary materials.

## Ethics statement

The studies involving human participants were reviewed and approved by Institutional Ethics Committee, Amrita Institute of Medical Science. The patients/participants provided their written informed consent to participate in this study.

## Author contributions

PB contributed to design of study, data collection, data interpretation, scale development, manuscript writing, manuscript revision, and final approval of the version to be published. VK contributed to design of study, statistical analysis, data interpretation, scale development, manuscript revision, and final approval of the version to be published. CJ contributed to conceptualization, design of study, data interpretation, supervision, scale development, manuscript revision, and final approval of the version to be published. All authors contributed to the article and approved the submitted version.

## Conflict of interest

The authors declare that the research was conducted in the absence of any commercial or financial relationships that could be construed as a potential conflict of interest.

## Publisher's note

All claims expressed in this article are solely those of the authors and do not necessarily represent those of their affiliated organizations, or those of the publisher, the editors and the reviewers. Any product that may be evaluated in this article, or claim that may be made by its manufacturer, is not guaranteed or endorsed by the publisher.
