# Supplementary material for: Development and validation of cultural competence assessment tool for healthcare professionals, India
Source: Front Public Health. 2022 Aug 23;10:919386. doi: 10.3389/fpubh.2022.919386 (PMC9445189; doi:10.3389/fpubh.2022.919386)
Supplement: Supplementary file 1 [file Data_Sheet_1.docx]

**Supplemental Table 1. Descriptive analysis of domains**

| **Domain** | **Mean + SD** | **Median** | **Interquartile Range** | | |
| --- | --- | --- | --- | --- | --- |
|  |  |  | **Q1** | **Q2** | **Q3** |
| Domain 1  Cultural awareness | 24.49±3.80 | 25 | 22 | 25 | 27 |
| Domain 2  Cultural sensitivity | 12.18±2.21 | 12 | 11 | 12 | 14 |
| Domain 3  Cultural knowledge | 14.01±2.20 | 14 | 13 | 14 | 16 |
| Domain 4  Cultural skills | 10.21±1.61 | 10 | 9 | 10 | 11 |
| Domain 5  Cultural behavior | 13.87±2.05 | 14 | 13 | 14 | 15 |
| Domain 6  Cultural desire | 15.57±2.19 | 16 | 14 | 16 | 16 |
| CC Scale total | 90.32±8.41 | 91 | 84 | 91 | 96 |

**Supplemental Table 2. – Estimated Floor and ceiling effect**

| **Domain** | **Ceiling(%)** | **Flooring(%)** | **N** |
| --- | --- | --- | --- |
| Domain 1  Cultural awareness | 12.41% | 10.7% | 67 |
| Domain 2  Cultural sensitivity | 11.72% | 12.76% | 71 |
| Domain 3  Cultural knowledge | 24.48% | 7.58% | 93 |
| Domain 4  Cultural skills | 5.52% | 14.14% | 57 |
| Domain 5  Cultural behavior | 5.86% | 11.72% | 51 |
| Domain 6  Cultural desire | 9.31% | 8.62% | 52 |
| CC Scale total | 12.41% | 11.38% | 69 |

**Supplemental Table 3. Test Re-test Reliability (Intra Class Correlation Coefficient) before item reduction (35 items)**

| **Sl.No.** | **Dimension** | **ICC** | **Lower Bound** | **Upper Bound** |
| --- | --- | --- | --- | --- |
| 1 | Overall | 0.640 | 0.244 | 0.829 |
| 2 | Cultural awareness | 0.546 | 0.052 | 0.783 |
| 3 | Cultural sensitivity | 0.704 | 0.374 | 0.860 |
| 4 | Cultural knowledge | 0.661 | 0.289 | 0.839 |
| 5 | Cultural skills | 0.184 | -0.541 | 0.571 |
| 6 | Cultural behavior | 0.583 | 0.121 | 0.802 |
| 7 | Cultural desire | 0.822 | 0.631 | 0.915 |

**Supplemental Table 4. Test Re-test Reliability (Intra Class Correlation Coefficient) after item reduction (27 items)**

| **Sl.No** | **Domains** | **ICC** | **Lower Bound** | **Upper Bound** |
| --- | --- | --- | --- | --- |
| **1** | Overall | 0.767 | 0.491 | 0.891 |
| **2** | Cultural awareness | 0.604 | 0.156 | 0.813 |
| **3** | Cultural sensitivity | 0.704 | 0.374 | 0.860 |
| **4** | Cultural knowledge | 0.773 | 0.521 | 0.892 |
| **5** | Cultural skills | 0.566 | 0.118 | 0.790 |
| **6** | Cultural behavior | 0.795 | 0.573 | 0.902 |
| **7** | Cultural desire | 0.822 | 0.631 | 0.915 |

**Supplemental Table 5. Table showing** **internal consistency using Cronbach’s alpha (before item deletion)**

| **Sl.No.** | **Dimension** | **Cronbach’s Alpha** | **No. of items** |
| --- | --- | --- | --- |
| 1 | Overall | 0.694 | 35 |
| 2 | Cultural awareness | 0.522 | 9 |
| 3 | Cultural sensitivity | 0.609 | 5 |
| 4 | Cultural knowledge | 0.421 | 6 |
| 5 | Cultural skills | 0.419 | 5 |
| 6 | Cultural behavior | 0.367 | 6 |
| 7 | Cultural desire | 0.818 | 4 |

**Supplemental Table 6. Table showing internal consistency using Cronbach’s alpha (before item deletion)**

| **Sl.No** | **Dimension** | **Items deleted** | **Cronbach’s Alpha** | **No. of items** |
| --- | --- | --- | --- | --- |
| 1 | Overall | B2, B4, D5, D4, E2, E3, F6, F4 | 0.700 | 27 |
| 2 | Cultural awareness | B2, B4 | 0.642 | 7 |
| 3 | Cultural sensitivity | No Deletion | 0.609 | 5 |
| 4 | Cultural knowledge | D5, D4 | 0.632 | 4 |
| 5 | Cultural skills | E2, E3 | 0.673 | 3 |
| 6 | Cultural behavior | F6, F4 | 0.683 | 4 |
| 7 | Cultural desire | No Deletion | 0.818 | 4 |

**Supplemental Table 7. Table showing results of test of reliability (Internal consistency)**

| **Domain** | **Cronbach’s Alpha** | **Lower Bound** | **Upper Bound** |
| --- | --- | --- | --- |
| Cultural awareness  (7 items) | 0.618 | 0.547 | 0.681 |
| Cultural sensitivity  (4 items) | 0.309 | 0.170 | 0.430 |
| Cultural knowledge  (4 items) | 0.579 | 0.494 | 0.653 |
| Cultural skills  (3 items) | -0.024 | -0.247 | 0.164 |
| Cultural behavior  (4 items) | 0.408 | 0.288 | 0.512 |
| Cultural desire  (4 items) | 0.798 | 0.758 | 0.834 |
| CC Scale total  (26 items) | 0.734 | 0.688 | 0.777 (Acceptable) |

**Supplemental Table 8. Table showing content validation of domains (Number of domains = 7)**

| **Domain** | **Expert 1** | **Expert 2** | **Expert 3** | **Expert 4** | **Expert 5** | **Expert 6** | **Relevant**  **(Score 2 or 3)** | **I-CVI** | **UA** | **Interpretation** |
| --- | --- | --- | --- | --- | --- | --- | --- | --- | --- | --- |
| Domain 1 | 3 | 3 | 2 | 2 | 3 | 3 | 6 | 1 | 1 | Appropriate |
| Domain 2 | 2 | 3 | 3 | 3 | 3 | 3 | 6 | 1 | 1 | Appropriate |
| Domain 3 | 2 | 3 | 3 | 2 | 3 | 3 | 6 | 1 | 1 | Appropriate |
| Domain 4 | 3 | 3 | 1 | 2 | 3 | 3 | 5 | 0.83 | 0 | Appropriate |
| Domain 5 | 1 | 3 | 3 | 2 | 2 | 2 | 5 | 0.83 | 0 | Appropriate |
| Domain 6 | 2 | 3 | 1 | 2 | 3 | 3 | 5 | 0.83 | 0 | Appropriate |
| Domain 7 | 1 | 3 | 1 | 3 | 3 | 1 | 3 | 0.5 | 0 | Eliminated/  Needs Revision |

S-CVI/Ave 0.86

S-CVI/UA 0.43

Fleiss Kappa 0.13(Poor)

Overall Percentage agreement 41.90%

**Supplemental Table 9. Table showing content validation of items (Number of items = 43)**

| **Item** | **Expert 1** | **Expert 2** | **Expert 3** | **Expert 4** | **Expert 5** | **Relevant  (Score 3 or 4)** | **I-CVI** | **UA** | **Interpretation** |
| --- | --- | --- | --- | --- | --- | --- | --- | --- | --- |
| **1** | 4 | 3 | 3 | 4 | 1 | 4 | 0.8 | 0 | Appropriate |
| **2** | 4 | 3 | 3 | 4 | 4 | 5 | 1 | 1 | Appropriate |
| **3** | 4 | 3 | 3 | 4 | 1 | 4 | 0.8 | 0 | Appropriate |
| **4** | 3 | 4 | 3 | 4 | 4 | 5 | 1 | 1 | Appropriate |
| **5** | 4 | 3 | 4 | 4 | 3 | 5 | 1 | 1 | Appropriate |
| **6** | 4 | 3 | 3 | 4 | 3 | 5 | 1 | 1 | Appropriate |
| **7** | 4 | 4 | 4 | 4 | 4 | 5 | 1 | 1 | Appropriate |
| **8** | 4 | 3 | 2 | 3 | 4 | 4 | 0.8 | 0 | Appropriate |
| **9** | 4 | 4 | 4 | 4 | 4 | 5 | 1 | 1 | Appropriate |
| **10** | 2 | 3 | 2 | 3 | 1 | 2 | 0.4 | 0 | Eliminated |
| **11** | 2 | 3 | 3 | 3 | 1 | 3 | 0.6 | 0 | Need for revision |
| **12** | 4 | 2 | 3 | 2 | 4 | 3 | 0.6 | 0 | Need for revision |
| **13** | 4 | 4 | 3 | 3 | 4 | 5 | 1 | 1 | Appropriate |
| **14** | 4 | 4 | 3 | 3 | 2 | 4 | 0.8 | 0 | Appropriate |
| **15** | 2 | 3 | 2 | 3 | 4 | 3 | 0.6 | 0 | Need for revision |
| **16** | 3 | 3 | 3 | 3 | 4 | 5 | 1 | 1 | Appropriate |
| **17** | 3 | 4 | 3 | 4 | 3 | 5 | 1 | 1 | Appropriate |
| **18** | 3 | 4 | 3 | 3 | 3 | 5 | 1 | 1 | Appropriate |
| **19** | 4 | 4 | 3 | 3 | 4 | 5 | 1 | 1 | Appropriate |
| **20** | 4 | 3 | 3 | 3 | 4 | 5 | 1 | 1 | Appropriate |
| **21** | 4 | 3 | 2 | 3 | 3 | 4 | 0.8 | 0 | Appropriate |
| **22** | 4 | 4 | 3 | 4 | 4 | 5 | 1 | 1 | Appropriate |
| **23** | 2 | 4 | 3 | 4 | 4 | 4 | 0.8 | 0 | Appropriate |
| **24** | 3 | 4 | 3 | 4 | 4 | 5 | 1 | 1 | Appropriate |
| **25** | 4 | 4 | 3 | 4 | 4 | 5 | 1 | 1 | Appropriate |
| **26** | 4 | 4 | 2 | 4 | 4 | 4 | 0.8 | 0 | Appropriate |
| **27** | 4 | 4 | 3 | 4 | 4 | 5 | 1 | 1 | Appropriate |
| **28** | 4 | 4 | 3 | 4 | 4 | 5 | 1 | 1 | Appropriate |
| **29** | 4 | 3 | 3 | 4 | 3 | 5 | 1 | 1 | Appropriate |
| **30** | 2 | 4 | 4 | 4 | 3 | 4 | 0.8 | 0 | Appropriate |
| **31** | 4 | 4 | 3 | 4 | 4 | 5 | 1 | 1 | Appropriate |
| **32** | 4 | 4 | 3 | 4 | 4 | 5 | 1 | 1 | Appropriate |
| **33** | 4 | 4 | 2 | 4 | 2 | 3 | 0.6 | 0 | Need for revision |
| **34** | 4 | 3 | 2 | 3 | 3 | 4 | 0.8 | 0 | Appropriate |
| **35** | 4 | 4 | 3 | 4 | 1 | 4 | 0.8 | 0 | Appropriate |
| **36** | 4 | 4 | 3 | 3 | 4 | 5 | 1 | 1 | Appropriate |
| **37** | 2 | 1 | 1 | 3 | 4 | 2 | 0.4 | 0 | Eliminated |
| **38** | 4 | 3 | 1 | 4 | 4 | 4 | 0.8 | 0 | Appropriate |
| **39** | 4 | 4 | 2 | 4 | 4 | 4 | 0.8 | 0 | Appropriate |
| **40** | 2 | 4 | 3 | 3 | 4 | 4 | 0.8 | 0 | Appropriate |
| **41** | 3 | 4 | 3 | 3 | 4 | 5 | 1 | 1 | Appropriate |
| **42** | 3 | 4 | 3 | 3 | 1 | 4 | 0.8 | 0 | Appropriate |
| **43** | 3 | 4 | 3 | 4 | 4 | 5 | 1 | 1 | Appropriate |
|  |  |  |  |  |  |  | 28.6 | 20 |  |

S-CVI/Ave 0.67

S-CVI/UA 0.47

Kappa 0.22

Score combined Kappa 0.57

**Supplemental Table 10. Table showing content validation of domains (after domain elimination)**

| **Domain** | **Expert 1** | **Expert 2** | **Expert 3** | **Expert 4** | **Expert 5** | **Expert 6** | **Relevant (Score 2 or 3)** | **I-CVI** | **UA** | **Interpretation** |
| --- | --- | --- | --- | --- | --- | --- | --- | --- | --- | --- |
| Domain 1 | 3 | 3 | 2 | 2 | 3 | 3 | 6 | 1 | 1 | Appropriate |
| Domain 2 | 2 | 3 | 3 | 3 | 3 | 3 | 6 | 1 | 1 | Appropriate |
| Domain 3 | 2 | 3 | 3 | 2 | 3 | 3 | 6 | 1 | 1 | Appropriate |
| Domain 4 | 3 | 3 | 1 | 2 | 3 | 3 | 5 | 0.83 | 0 | Appropriate |
| Domain 5 | 1 | 3 | 3 | 2 | 2 | 2 | 5 | 0.83 | 0 | Appropriate |
| Domain 6 | 2 | 3 | 1 | 2 | 3 | 3 | 5 | 0.83 | 0 | Appropriate |

S-CVI/Ave 0.92

S-CVI/UA 0.5

Fleiss Kappa 0.67(Fair)

Overall Percentage agreement 83.30%

**Supplemental Table 11. Table showing content validation of items (after item deletion)**

| **Item** | **Expert 1** | **Expert 2** | **Expert 3** | **Expert 4** | **Expert 5** | **Relevant**  **(Score 2 or 3)** | **I-CVI** | **UA** | **Interpretation** |
| --- | --- | --- | --- | --- | --- | --- | --- | --- | --- |
| **1** | 4 | 3 | 3 | 4 | 1 | 4 | 0.8 | 0 | Appropriate |
| **2** | 4 | 3 | 3 | 4 | 4 | 5 | 1 | 1 | Appropriate |
| **3** | 4 | 3 | 3 | 4 | 1 | 4 | 0.8 | 0 | Appropriate |
| **4** | 3 | 4 | 3 | 4 | 4 | 5 | 1 | 1 | Appropriate |
| **5** | 4 | 3 | 4 | 4 | 3 | 5 | 1 | 1 | Appropriate |
| **6** | 4 | 3 | 3 | 4 | 3 | 5 | 1 | 1 | Appropriate |
| **7** | 4 | 4 | 4 | 4 | 4 | 5 | 1 | 1 | Appropriate |
| **8** | 4 | 3 | 2 | 3 | 4 | 4 | 0.8 | 0 | Appropriate |
| **9** | 4 | 4 | 4 | 4 | 4 | 5 | 1 | 1 | Appropriate |
| **10** | 2 | 3 | 3 | 3 | 1 | 3 | 0.6 | 0 | Need for revision |
| **11** | 4 | 2 | 3 | 2 | 4 | 3 | 0.6 | 0 | Need for revision |
| **12** | 4 | 4 | 3 | 3 | 4 | 5 | 1 | 1 | Appropriate |
| **13** | 4 | 4 | 3 | 3 | 2 | 4 | 0.8 | 0 | Appropriate |
| **14** | 2 | 3 | 2 | 3 | 4 | 3 | 0.6 | 0 | Need for revision |
| **15** | 3 | 3 | 3 | 3 | 4 | 5 | 1 | 1 | Appropriate |
| **16** | 3 | 4 | 3 | 4 | 3 | 5 | 1 | 1 | Appropriate |
| **17** | 3 | 4 | 3 | 3 | 3 | 5 | 1 | 1 | Appropriate |
| **18** | 4 | 4 | 3 | 3 | 4 | 5 | 1 | 1 | Appropriate |
| **19** | 4 | 3 | 3 | 3 | 4 | 5 | 1 | 1 | Appropriate |
| **20** | 4 | 3 | 2 | 3 | 3 | 4 | 0.8 | 0 | Appropriate |
| **21** | 4 | 4 | 3 | 4 | 4 | 5 | 1 | 1 | Appropriate |
| **22** | 2 | 4 | 3 | 4 | 4 | 4 | 0.8 | 0 | Appropriate |
| **23** | 3 | 4 | 3 | 4 | 4 | 5 | 1 | 1 | Appropriate |
| **24** | 4 | 4 | 3 | 4 | 4 | 5 | 1 | 1 | Appropriate |
| **25** | 4 | 4 | 2 | 4 | 4 | 4 | 0.8 | 0 | Appropriate |
| **26** | 4 | 4 | 3 | 4 | 4 | 5 | 1 | 1 | Appropriate |
| **27** | 4 | 4 | 3 | 4 | 4 | 5 | 1 | 1 | Appropriate |
| **28** | 4 | 3 | 3 | 4 | 3 | 5 | 1 | 1 | Appropriate |
| **29** | 2 | 4 | 4 | 4 | 3 | 4 | 0.8 | 0 | Appropriate |
| **30** | 4 | 4 | 3 | 4 | 4 | 5 | 1 | 1 | Appropriate |
| **31** | 4 | 4 | 3 | 4 | 4 | 5 | 1 | 1 | Appropriate |
| **32** | 4 | 4 | 2 | 4 | 2 | 3 | 0.6 | 0 | Need for revision |
| **33** | 4 | 3 | 2 | 3 | 3 | 4 | 0.8 | 0 | Appropriate |
| **34** | 4 | 4 | 3 | 4 | 1 | 4 | 0.8 | 0 | Appropriate |
| **35** | 4 | 4 | 3 | 3 | 4 | 5 | 1 | 1 | Appropriate |

S-CVI/Ave 0.87

S-CVI/UA 0.58

Kappa 0.25

Score combined Kappa 0.72

Percentage agreement 81.43%

Number of Items: 35

**Supplemental Table 12. . Domains identified in factor analysis**

| **Identified Domain** | **Description** |
| --- | --- |
| Domain 1  Cultural awareness | Recognition and acceptance of one’s own cultural background with the perception of cultural differences in others. It is an appreciation that personal views influence cultural perspectives, personal prejudice and personal values. |
| Domain 2  Cultural sensitivity | It is the awareness that cultural differences and similarities between people exist, without assigning them a value – positive or negative, better or worse, right or wrong. |
| Domain 3  Cultural knowledge | It is the process of seeking and obtaining a sound educational foundation about diverse cultural and ethnic groups. |
| Domain 4  Cultural skills | It is the application of cultural knowledge to clinical care. |
| Domain 5  Cultural behavior | It is the behavior that occurs within a specific culture. Certain behavior may only be specific to a single culture. |
| Domain 6  Cultural desire | It means the motivation of health care provider to’ want to’, rather than ‘have to’, engage in the process of becoming culturally aware, culturally knowledgeable, culturally skillful, and familiar with cultural encounters. |

**Supplemental Table 13. Final rotated factor loading matrix after PCA and Varimax rotation**

| **Items selected after factor rotations** | **Factor 1:**  **Culture Awareness** | **Factor 2:**  **Culture**  **Sensitivity** | **Factor 3:**  **Culture**  **Knowledge** | **Factor 4:**  **Culture**  **Skills** | **Factor 5:**  **Culture**  **Behavior** | **Factor 6:**  **Culture**  **Desire** |
| --- | --- | --- | --- | --- | --- | --- |
| **Item B1** | 0.671 | -0.132 | -0.352 | 0.174 | 0.232 | 0.457 |
| **Item B2** | 0.375 | -0.066 | -0.426 | -0.111 | 0.350 | 0.241 |
| **Item B3** | 0.649 | 0.122 | 0.004 | -0.040 | 0.060 | -0.027 |
| **Item B4** | 0.429 | -0.019 | 0.142 | 0.275 | 0.241 | -0.191 |
| **Item B5** | 0.713 | 0.162 | 0.028 | 0.277 | 0.179 | -0.103 |
| **Item B6** | 0.589 | -0.069 | 0.080 | 0.077 | 0.142 | 0.117 |
| **Item B7** | 0.467 | -0.279 | 0.354 | 0.226 | 0.147 | -0.005 |
| **Item C1** | -0.030 | 0.276 | -0.289 | -0.228 | -0.553 | -0.081 |
| **Item C2** | 0.221 | 0.693 | -0.189 | 0.047 | 0.010 | 0.066 |
| **Item C3** | 0.203 | 0.535 | 0.144 | -0.151 | -0.680 | 0.090 |
| **Item C4** | -0.009 | 0.460 | -0.189 | 0.204 | 0.429 | 0.245 |
| **Item C5** | 0.013 | 0.038 | -0.190 | -0.607 | 0.009 | -0.257 |
| **Item D1** | 0.211 | -0.049 | 0.490 | 0.313 | 0.040 | 0.163 |
| **Item D2** | 0.178 | 0.017 | 0.756 | -0.062 | 0.147 | 0.030 |
| **Item D3** | 0.161 | 0.241 | 0.662 | -0.019 | 0.171 | 0.120 |
| **Item D4** | 0.250 | 0.179 | 0.404 | 0.301 | -0.141 | 0.037 |
| **Item E1** | -0.023 | 0.066 | 0.087 | 0.087 | -0.764 | -0.085 |
| **Item E2** | 0.166 | 0.005 | 0.338 | 0.436 | 0.046 | 0.016 |
| **Item E3** | 0.354 | -0.043 | 0.221 | 0.463 | -0.047 | 0.141 |
| **Item F1** | 0.165 | 0.087 | 0.188 | -0.061 | 0.529 | 0.065 |
| **Item F2** | 0.136 | 0.189 | 0.396 | 0.116 | 0.433 | -0.211 |
| **Item F3** | 0.267 | -0.253 | -0.067 | -0.064 | 0.303 | -0.166 |
| **Item F4** | 0.262 | 0.053 | 0.084 | 0.262 | 0.542 | -0.240 |
| **Item G1** | -0.045 | 0.274 | 0.158 | 0.256 | 0.079 | 0.644 |
| **Item G2** | 0.082 | 0.037 | 0.159 | -0.030 | 0.146 | 0.766 |
| **Item G3** | 0.072 | 0.165 | 0.218 | 0.155 | 0.182 | 0.664 |
| **Item G4** | 0.242 | 0.079 | 0.190 | -0.041 | -0.078 | 0.689 |
| Eigen Values of each factor | 4.98 | 2.45 | 1.73 | 1.52 | 1.43 | 1.21 |
| % Variance explained by each factor | 11.47% | 9.17% | 7.96% | 7.46% | 7.14% | 6.14% |
| Cumulative % variance explained by the tool | 49.35% |  |  |  |  |  |

**Supplemental Table 14. Final rotated factor loading matrix after PCA and Varimax rotation (after item reduction)**

| **Items selected after factor rotations** | **Factor 1:**  **Cultural Awareness** | **Factor 2:**  **Cultural**  **Sensitivity** | **Factor 3:**  **Cultural**  **Knowledge** | **Factor 4:**  **Cultural**  **Skills** | **Factor 5:**  **Cultural**  **Behavior** | **Factor 6:**  **Cultural**  **Desire** |
| --- | --- | --- | --- | --- | --- | --- |
| **Item B1** | 0.671 | -0.132 | -0.352 | 0.174 | 0.232 | 0.457 |
| **Item B2** | 0.409 | -0.066 | -0.426 | -0.111 | 0.350 | 0.241 |
| **Item B3** | 0.670 | 0.122 | 0.004 | -0.040 | 0.060 | -0.027 |
| **Item B4** | 0.420 | -0.019 | 0.142 | 0.275 | 0.241 | -0.191 |
| **Item B5** | 0.624 | 0.162 | 0.028 | 0.277 | 0.179 | -0.103 |
| **Item B6** | 0.628 | -0.069 | 0.080 | 0.077 | 0.142 | 0.117 |
| **Item B7** | 0.637 | -0.279 | 0.354 | 0.226 | 0.147 | -0.005 |
| **Item C1** | Item eliminated | | | | | |
| **Item C2** | 0.221 | 0.458 | -0.189 | 0.047 | 0.010 | 0.066 |
| **Item C3** | 0.203 | 0.535 | 0.144 | -0.151 | -0.680 | 0.090 |
| **Item C4** | -0.009 | 0.544 | -0.189 | 0.204 | 0.375 | 0.245 |
| **Item C5** | 0.013 | 0.473 | -0.190 | -0.607 | 0.009 | -0.257 |
| **Item D1** | 0.211 | -0.049 | 0.473 | 0.313 | 0.040 | 0.163 |
| **Item D2** | 0.178 | 0.017 | 0.756 | -0.062 | 0.147 | 0.030 |
| **Item D3** | 0.161 | 0.241 | 0.662 | -0.019 | 0.171 | 0.120 |
| **Item D4** | 0.250 | 0.179 | 0.507 | 0.301 | -0.141 | 0.037 |
| **Item E1** | -0.023 | 0.066 | 0.087 | 0.612 | -0.764 | -0.085 |
| **Item E2** | 0.166 | 0.005 | 0.338 | 0.425 | 0.046 | 0.016 |
| **Item E3** | 0.354 | -0.043 | 0.221 | 0.468 | -0.047 | 0.141 |
| **Item F1** | 0.165 | 0.087 | 0.188 | -0.061 | 0.384 | 0.065 |
| **Item F2** | 0.136 | 0.189 | 0.396 | 0.116 | 0.426 | -0.211 |
| **Item F3** | 0.267 | -0.253 | -0.067 | -0.064 | 0.303 | -0.166 |
| **Item F4** | 0.262 | 0.053 | 0.084 | 0.262 | 0.531 | -0.240 |
| **Item G1** | -0.045 | 0.274 | 0.158 | 0.256 | 0.079 | 0.679 |
| **Item G2** | 0.082 | 0.037 | 0.159 | -0.030 | 0.146 | 0.658 |
| **Item G3** | 0.072 | 0.165 | 0.218 | 0.155 | 0.182 | 0.714 |
| **Item G4** | 0.242 | 0.079 | 0.190 | -0.041 | -0.078 | 0.597 |
| Eigen Values of each factor | 4.98 | 2.45 | 1.73 | 1.52 | 1.43 | 1.21 |
| % Variance explained by each factor | 10.49% | 9.60% | 8.71% | 8.09% | 7.58% | 5.89% |
| Cumulative % variance explained by the tool | 50.36% |  |  |  |  |  |
